# An Efficient Electrochemical Sensor Based on NiCo_2_O_4_ Nanoplates and Ionic Liquid for Determination of Favipiravir in the Presence of Acetaminophen

**DOI:** 10.3390/bios13080814

**Published:** 2023-08-14

**Authors:** Somayeh Tajik, Fatemeh Sharifi, Behnaz Aflatoonian, Sayed Zia Mohammadi

**Affiliations:** 1 Research Center of Tropical and Infectious Diseases, Kerman University of Medical Sciences, Kerman P.O. Box 76169-13555, Iran; fatemeh7267@gmail.com (F.S.); aflatoonianbehnaz@gmail.com (B.A.); 2 Department of Chemistry, Payame Noor University, Tehran P.O. Box 19395-3697, Iran; szmohammadi@pnu.ac.ir

**Keywords:** carbon paste electrode, NiCo_2_O_4_ nanoplates, 1-hexyl-3-methylimidazolium tetrafluoroborate, favipiravir, acetaminophen

## Abstract

Based on the modification of carbon paste electrode with NiCo_2_O_4_ nanoplates and 1-hexyl-3-methylimidazolium tetrafluoroborate, a new electrochemical sensing platform for the sensing of favipiravir (a drug with potential therapeutic efficacy in treating COVID-19 patients) in the presence of acetaminophen was prepared. For determining the electrochemical behavior of favipiravir, cyclic voltammetry, differential pulse voltammetry, and chronoamperometry have been utilized. When compared to the unmodified carbon paste electrode, the results of the cyclic voltammetry showed that the proposed NiCo_2_O_4_ nanoplates/1-hexyl-3-methylimidazolium tetrafluoroborate/carbon paste electrode had excellent catalytic activity for the oxidation of the favipiravir in phosphate buffer solution (pH = 7.0). This was due to the synergistic influence of 1-hexyl-3-methylimidazolium tetrafluoroborate (ionic liquid) and NiCo_2_O_4_ nanoplates. In the optimized conditions of favipiravir measurement, NiCo_2_O_4_ nanoplates/1-hexyl-3-methylimidazolium tetrafluoroborate/carbon paste electrode had several benefits, such as a wide dynamic linear between 0.004 and 115.0 µM, a high sensitivity of 0.1672 µA/µM, and a small limit of detection of 1.0 nM. Furthermore, the NiCo_2_O_4_ nanoplates/1-hexyl-3-methylimidazolium tetrafluoroborate/carbon paste electrode sensor presented a good capability to investigate the favipiravir and acetaminophen levels in real samples with satisfactory recoveries.

## 1. Introduction

Since December 2019, a pandemic caused by the 2019 coronavirus illness (COVID-19) has rapidly spread throughout the world. Since the start of the pandemic, COVID-19 has claimed millions of lives. Its severe acute respiratory syndrome can be fatal and affects the lower respiratory system. Numerous approved vaccines fell short in their attempts to halt the pandemic’s deadly spread. This may be due to the inefficiency and unavailability of vaccination and mutation treatments, as well as a lack of suitable substitute therapy techniques [1,2,3,4]. Repurposing the use of commercially accessible antiviral medications such as favipiravir (FVP) is therefore seen as a workable and successful approach.

The Fujifilm Toyama Chemical Company in Japan created the purine nucleic acid analogue FVP (trade name: Avigan) [5]. FVP functions as a prodrug that is intracellularly transformed into its active metabolite, FVP-RTP (ribofuranosyl 5′-triphosphate) [6,7,8]. Numerous additional RNA viruses can be inhibited by the FVP-RTP molecule. Although the precise mechanism of action is uncertain, it is hypothesised that FVP-RTP may accidentally incorporate into a viral RNA chain as it grows or may bind to conserved polymerase domains to stop viral RNA replication [9,10]. Either chain termination or deadly mutagenesis by ambiguous base pairing could result from the inclusion of FVP-RTP in the developing viral RNA. The effectiveness of FVP has been tested against a number of RNA viruses due to its activity against RNA viruses, such as Influenza A virus subtype H1N1, SARS-CoV-2, Ebola virus, etc. [11,12,13]. FVP was approved for the treatment of the illness during the 2014 influenza pandemic in Japan and had a strong potential for in vitro action against the acute respiratory syndrome coronavirus [7]. To facilitate pharmacokinetic and metabolic research using biological samples from both humans and animals, as well as to regularly check the quality of pharmaceutical formulations, it is imperative to develop suitable analytical methods for the identification of effective antiviral agents for COVID-19, including FVP. In addition to meeting these standards, this biological matrix research will aid in the creation of new medications.

Acetaminophen (AC), commonly referred to as paracetamol, is a febrile analgesic that also treats osteoarthritis, headaches, backaches, and post-operative pain [14]. Both inhibiting prostaglandin production in the central nervous system (CNS) and anaesthetizing the hypothalamic thermoregulatory centre allow paracetamol to fulfil these potentials [15]. Since the liver is where AC is primarily processed, many studies have demonstrated that taking too much of it can cause abrupt liver failure [16]. For COVID-19 patients, AC was frequently given as a first-line antipyretic and analgesic without any consideration for the potential risk of associated toxicities [17]. The most frequent clinical signs of COVID-19 include fever, exhaustion, dry cough, and pulmonary abnormalities. Hence, over-the-counter (OTC) medications such as non-steroidal anti-inflammatory drugs (NSAIDs) and AC that relieve fever and pain may catch people’s attention [18,19]. The COVID-19 Treatment Guidelines Panel of Many Countries covered FVP and AC. Therefore, this study’s objective is to develop a method of analysis for FVP and AC determination in actual samples.

Therefore, it is essential to develop an analytical method that is sensitive, reliable, quick, and affordable to trace and ascertain FVP and AC. The determination of FVP and AC has been conducted using a variety of methods, including chromatography [20,21,22], chemiluminescence [23], spectrofluorometry [24,25,26], and electrochemical methods [27,28,29]. The electrochemical methods stand out among them for their strong biological and environmental media analysis skills due to certain amazing traits such as affordability, usability, sensitivity, lack of toxicity, mobility, real-time analysis, and environmentally friendly practises [30,31,32,33,34,35,36,37,38,39,40]. The electrode surface modification has a substantial effect on how well the electrochemical method works. One of the main issues with the development of electrochemical sensors is the high overvoltage of compounds. The use of chemically modified electrodes in electroanalysis has a number of benefits, including a reduction in peak potential and an enhancement in sensitivity and selectivity [41,42,43,44,45,46].

In the development of electrochemical sensors, carbon paste electrodes (CPEs) have served a variety of purposes by providing mechanical stability, improved sensitivity and selectivity, and the utilisation of less valuable materials [47,48]. The CPE is an electrode made by uniformly mixing a binder and conductive carbon powder mixture and pouring it into an electrode tube [49]. Due to its low cost, ease of electrode surface renewal, simplicity, wide potential range, low background current, and remarkable adaptability, it is frequently employed in electrochemical studies [50,51,52,53,54]. CPEs are particularly desirable for modifying an electrode material with the addition of additional compounds, giving the electrode a set of preset characteristics. One or more components can be added to carbon paste to create a chemically modified CPE.

Ionic liquids (ILs) and nanomaterials are useful modifiers in this regard. Due to their exceptional qualities, including specific catalytic characteristics, broad electrochemical windows, low vapour pressures, excellent conductivities, and great thermal and chemical stability, ILs have recently gained a lot of attention [55,56].

Over the past few years, nanomaterials and their applications in numerous industries have emerged as a separate and active area of scientific and technical advancement [57,58,59,60,61,62,63,64,65]. Nanomaterials have transformed science, with their distinctive features enhancing the functionality of numerous systems and making them the preferred material in many areas. The development of nanomaterials has proven fundamental for the development of effective and efficient electrochemical sensors to be used in different application fields such as biomedical, environmental, and food analysis [66,67,68,69]. Due to their admirable physicochemical characteristics, the development of sensors using transition metal oxide nanostructures has shown promise. Binary metal oxides are better suited than transition metal oxides due to their excellent electrochemical properties and strong electrical conductivity [70,71,72]. Transition binary metal oxides, in particular cobalt-based spinel oxides, have a variety of oxidation states, are simple to synthesise, exhibit high durability in alkaline electrolytes, are low toxic, and are economically advantageous mixed-valence oxides [73,74,75]. Due to their eco-friendliness, low cost, and strong electrical conductivity, NiCo_2_O_4_ nanoparticles with various shapes and morphologies have greatly attracted interest as prospective electrochemical electrodes in various electrochemical devices such as sensors and biosensors [76,77,78]. Moreover, NiCo_2_O_4_’s numerous oxidation states provide a high level of electronic conductivity and can be tuned to control both the electrochemical reactions and the electrochemical performances [79]. NiCo_2_O_4_ is used as an electrochemical material because of the benefits already mentioned, but it still has to be improved due to its low surface area, large pore volume, and weak inherent electronic conductivity [80]. Due to the advantages of the redox process with its large pore size, high surface area, short pathways, and rapid reaction kinetics, adopting materials with a two-dimensional or sheet-like shape can help prevent such bottlenecks [81,82]. An interesting electrode material to realise its application in chemical sensors might be NiCo_2_O_4_ with a 2D or sheet shape. As a result, NiCo_2_O_4_ with a 2D or sheet morphology may be a promising electrode material for chemical sensors.

In the current study, we tried to create a new electrochemical sensing platform by modifying a CPE with 1-hexyl-3-methylimidazolium tetrafluoroborate (HMIM BF_4_) and NiCo_2_O_4_ nanoplates (NiCo_2_O_4_ NPs) in order to analyse FVP and AC at the same time. The suggested sensor displayed greater selectivity, a lower limit of detection, amazing sensitivity, and wider dynamic linear ranges. Additionally, the practical applicability of our sensor was explored by sensing the FVP and AC in real specimens. The novelty of this work lies in the application of NiCo_2_O_4_ NPs/HMIM BF_4_/CPE as a sensing platform, which enabled the electrochemical detection of FVP in the presence of AC.

## 2. Experimental

### 2.1. Equipment and Materials

All electrochemical analyses have been conducted at ambient temperature using an Autolab PGSTAT 320 N Potentiostat/Galvanostat Analyzer (Utrecht, The Netherlands) with GPES (General Purpose Electrochemical System, version 4.9) software. In this investigation, a typical three-electrode cell at a temperature of 25 ± 1 °C was employed. It included a platinum wire as a counter electrode (Azar Electrode, Urmia, Iran), NiCo_2_O_4_ NPs/HMIM BF_4_/CPE as a working electrode, and Ag/AgCl/KCl (3.0 M) as a reference electrode (Azar Electrode, Urmia, Iran). To measure the pH of the solutions, a Switzerland-made Metrohm 713 pH meter with a glass electrode was used. All of the solutions were freshly prepared using Direct-Q^®^ 8 UV deionized water (Millipore, Burlington, MA, USA). For FE-SEM analysis, a MIRA3 scanning electron microscope (Tescan, Brno, Czech Republic) has been used.

All of the chemicals and solvents used in our protocol were of analytical grade and came from Merck and Sigma-Aldrich. Phosphoric acid has been utilized to make phosphate buffer solution (PBS), which was then pH-adjusted using NaOH.

### 2.2. Preparation of NiCo_2_O_4_ NPs

For the preparation of NiCo_2_O_4_ NPs, Ni(NO_3_)_2._6H_2_O (0.5 mmol, 0.145 gr), Co(NO_3_)_2._6H_2_O (1 mmol, 0.291 gr), NH_4_F (3 mmol, 0.111 gr), and urea (7.5 mmol, 0.45 gr) have been dispersed in deionized water (40 mL). After stirring for 40 min to create a clear pink solution, the prepared solution was placed in a Teflon-lined stainless-steel autoclave for three hours at 120 °C. After being cooled to laboratory temperature, the collected precipitate was thoroughly washed with deionized water and oven-dried for 12 h at 65 °C. The prepared product was then annealed for 150 min at 350 °C.

**Figure 1:**
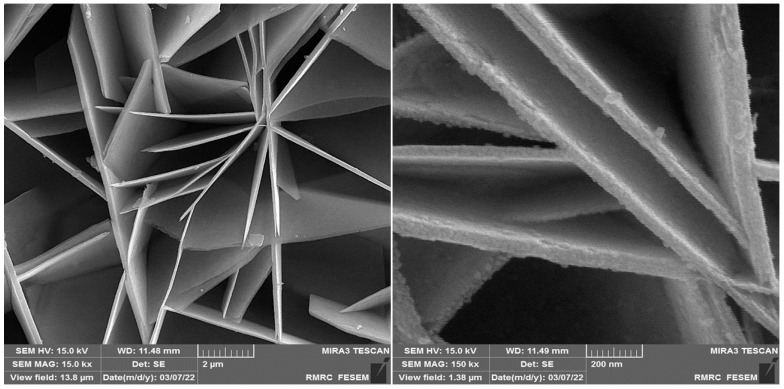
FE-SEM images of NiCo_2_O_4_ NPs.

The characterization of NiCo_2_O_4_ NPs has been reported in our previous work [83]. Figure 1 shows the FE-SEM images of NiCo_2_O_4_ NPs.

### 2.3. Preparation and Surface Modification of Electrode

In order to prepare NiCo_2_O_4_ NPs/HMIM BF_4_/CPE, 100 mg of NiCo_2_O_4_ NPs and 900 mg of graphite powder were combined in a mortar and then mixed for 10 min with 0.6 mL of paraffin oil and 0.2 mL of HMIM BF_4_ ionic liquid. The paste was dispensed into a glass tube to the required level, and a copper wire was positioned over the paste to make electrical contact. Finally, the surface of the electrode was polished on weighing paper to give it a smooth aspect before use.

In addition, NiCo_2_O_4_ NPs/CPE (without the use of HMIM BF_4_), HMIM BF_4_/CPE (without the use of NiCo_2_O_4_ NPs), and an unmodified CPE without the usage of HMIM BF_4_ and NiCo_2_O_4_ NPs were built for comparison.

### 2.4. Preparation of Real Samples

Five tablets of the FVP (labelled value of FVP = 200 mg per tablet) and five tablets of the AC (labelled value of AC = 500 mg per tablet) purchased from a local pharmacy in Kerman (Iran) were finely powdered in a mortar and pestle. Then, an accurately weighed amount of the homogenized FVP and AC powders was transferred into 100 mL of 0.1 M PBS (pH 7.0). The contents of the flasks were sonicated for 20 min to achieve complete dissolution. Finally, the solutions were filtered, and a suitable aliquot of the clear filtrate was collected. Afterward, we poured a determined volume of the transparent filtrates (4.7 µL of FVP and 2.5 µL of AC) into the electrochemical cell, consisting of 25 mL of 0.1 mol/L PBS (pH = 7.0), to record the differential pulse voltammograms.

## 3. Results and Discussion

### 3.1. Electrochemical Behaviour of FVP on NiCo_2_O_4_ NPs/HMIM BF_4_/CPE

The solution pH affected how FVP responded electrochemically. To ascertain the electrocatalytic oxidation of FVP, it appears essential to optimize the pH of the solution. Consequently, differential pulse voltammetry (DPV) investigated the effect of pH throughout a range of 2.0 to 9.0 on the FVP electro-oxidation on the NiCo_2_O_4_ NPs/HMIM BF_4_/CPE surface. At neutral conditions (pH = 7.0), the electrocatalytic FVP oxidation on the NiCo_2_O_4_ NPs/HMIM BF_4_/CPE surface was greatest (optimal).

**Figure 2:**
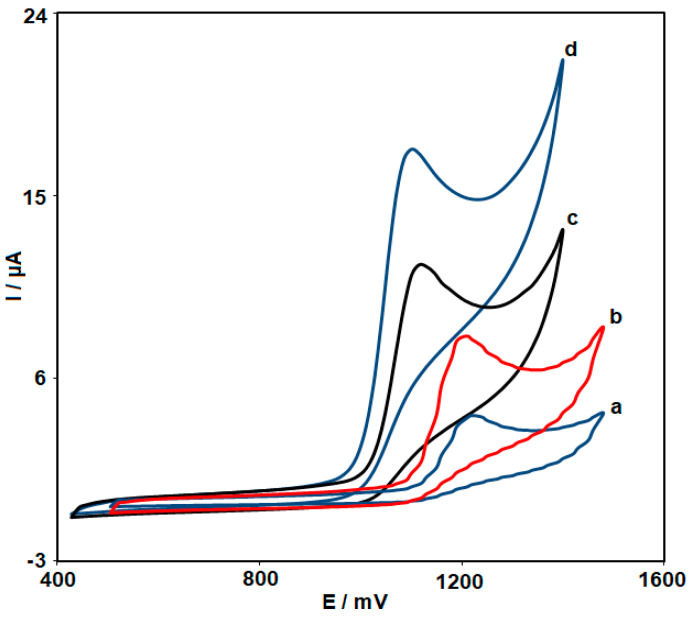
CV response of 100.0 μM FVP at (a) unmodified CPE, (b) NiCo_2_O_4_ NPs/CPE, (c) HMIM BF_4_/CPE, and (d) NiCo_2_O_4_ NPs/HMIM BF_4_/CPE in 0.1 M PBS of pH 7.0.

To demonstrate the enhancement effects of NiCo_2_O_4_ NPs and HMIM BF_4_, the electrochemical response of FVP was carried out by cyclic voltammetry (CV) at the different electrodes (Figure 2). Figure 2 shows the CVs of FVP (100.0 μM) at unmodified CPE (Curve a), NiCo_2_O_4_ NPs/CPE (Curve b), HMIM BF_4_/CPE (Curve c), and NiCo_2_O_4_ NPs/HMIM BF_4_/CPE (Curve d). Figure 2 shows the anodic peak potential at 1100 mV for FVP on the NiCo_2_O_4_ NPs/HMIM BF_4_/CPE surface and 1240 mV for FVP oxidation on the unmodified CPE surface (curve a). The graphs show that peak potential FVP oxidation on the NiCo_2_O_4_ NPs/HMIM BF_4_/CPE shifted from 1240 mV to negative values in contrast to the surface of the unmodified CPE. Regarding the FVP oxidation on the surface of NiCo_2_O_4_ NPs/CPE (curve b) and NiCo_2_O_4_ NPs/HMIM BF_4_/CPE (curve d), the anodic peak current was enhanced on NiCo_2_O_4_ NPs/HMIM BF_4_/CPE in comparison to NiCo_2_O_4_ NPs/CPE, which indicates that the presence of IL in the CPE caused the peak currents to be higher. These results correctly depict the enhancement effects caused by the presence of HMIM BF_4_ and NiCo_2_O_4_ NPs and also amplified the sensitivity of the electrode toward the oxidation of FVP.

### 3.2. Effect of Scan Rate

**Figure 3:**
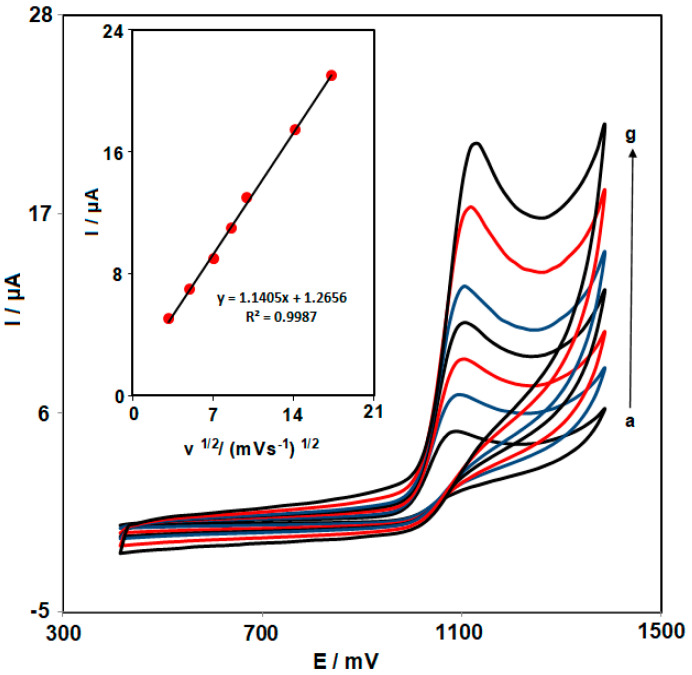
CV responses of 50.0 μM FVP in 0.1 M PBS (pH 7.0) at a scan rate of 10 to 300 mV s^−1^ at NiCo_2_O_4_ NPs/HMIM BF_4_/CPE (a: 10, b: 25, c: 50, d: 75, e: 100, f: 200, and g: 300 mV s^−1^). Inset: Plot of the oxidation peak current of FVP vs. v^1/2^.

Figure 3 depicts how different scan rates affect the FVP oxidation currents. The results show that raising the scan rate increases the peak currents. The diffusion-controlled nature of the oxidation processes can be confirmed by the linear plot of Ip versus v^1/2^ (the square root of the potential scan rate) for the analyte. It means that FVP reaches the electrode by diffusion, and after the oxidation process, the product of the oxidation also gets away from the electrode surface by diffusion.

### 3.3. Chronoamperometric Analysis

**Figure 4:**
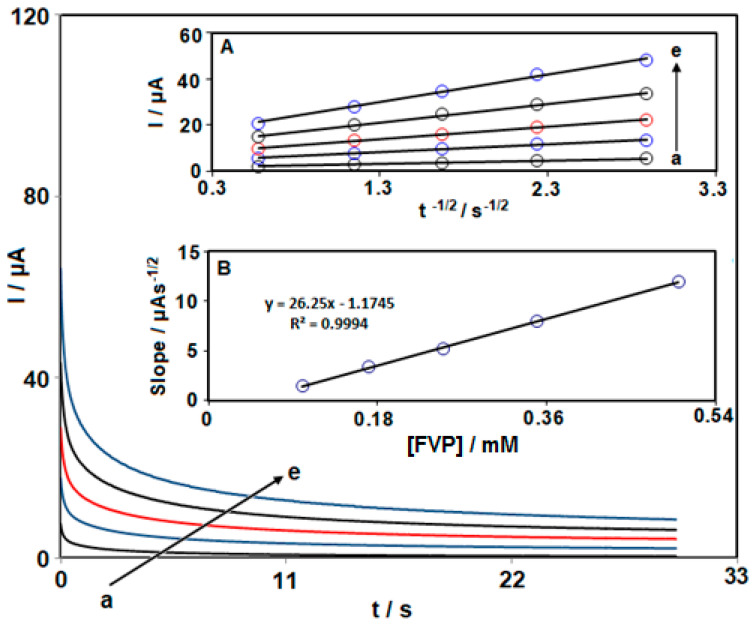
Single-step chronoamperograms obtained at NiCo_2_O_4_ NPs/HMIM BF_4_/CPE at various FVP concentrations (a: 0.1 mM, b: 0.17 mM, c: 0.25 mM, d: 0.35 mM, e: 0.5 mM) in 0.1 M PBS (pH 7.0). Insets: I vs. t^−1/2^ variation data from the chronoamperograms (**A**) and slope plot of the straight line vs. concentration of FVP (**B**).

In order to investigate FVP oxidation on the modified electrode, chronoamperometry was used. On the surface of NiCo_2_O_4_ NPs/HMIM BF_4_/CPE, FVP contents were determined by chronoamperometry using an 1150 mV working electrode potential. Additionally, the FVP’s diffusion coefficient (D) was determined. As shown in Figure 4 (Inset A), we showed the best-fitting I against t^−1/2^ plots for various FVP contents. The slopes from the straight lines have been subsequently plotted against various FVP contents, as shown in Figure 4 (Inset B). Based on this slope and Cottrell’s equation:

I = nFAD^1/2^C_b_ π^−1/2^ t^−1/2^

In this equation, n denotes the quantity of electrons moved (n = 2 according to the previous works such as reference [84]), F for Faraday’s constant, A for electrode surface area (cm^2^), C_b_ for the bulk concentration (mol cm^−3^), and t for time (s). For FVP, the average D value was determined to be ~7.1 × 10^−6^ cm^2^/s.

### 3.4. Quantitative Determination of FVP by DPV

**Figure 5:**
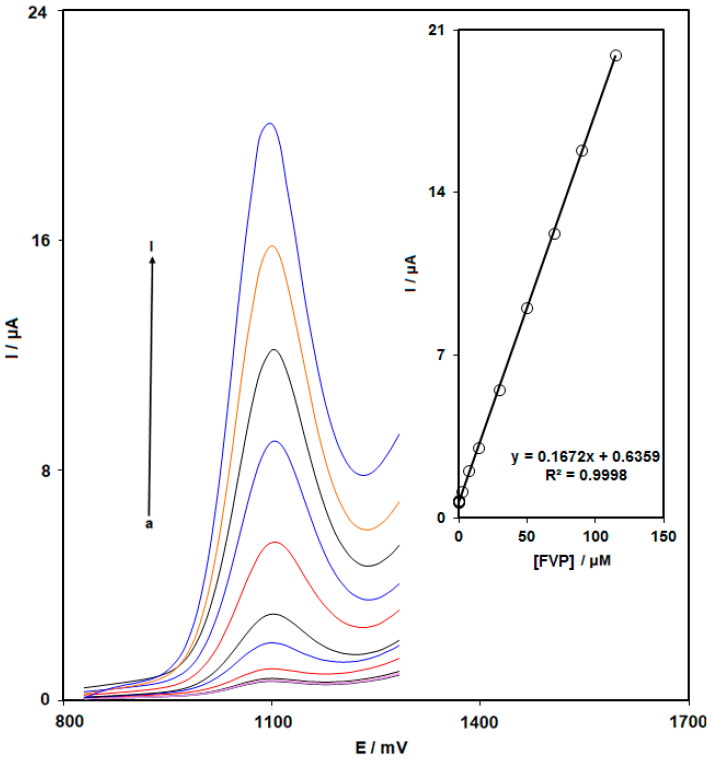
DPV response at NiCo_2_O_4_ NPs/HMIM BF_4_/CPE in 0.1 M PBS (pH = 7.0) with different concentrations of FVP (a: 0.004, b: 0.04, c: 0.1, d: 0.5, e: 2.5, f: 7.5, g: 15.0, h: 30.0, i: 50.0, j: 70.0, k: 90.0 and l: 115.0 μM). Inset: linear plot for the oxidation current response of FVP vs. its concentrations.

Under optimized experimental conditions, DPV analysis was carried out for varied FVP levels to investigate the limit of detection (LOD), linear dynamic range, and sensitivity of the NiCo_2_O_4_ NPs/HMIM BF_4_/CPE (Figure 5). As anticipated, the increased FVP level increased the peak current. The results in Figure 5 (Inset) showed a linear relationship between the FVP peak currents as well as its concentrations of 0.004 to 115.0 μM, using the linear regression equation of I_pa_ (μA) = 0.1672C_FVP_ + 0.7182 (R^2^ = 0.999) and the sensitivity of 0.1672 μA/μM. In addition, the detection limit, C_m_, of FVP has been determined by the following equation:

C_m_ = 3S_b_/m

where m is the calibration plot’s slope (0.1672 μA μM^–1^), and S_b_ is the blank response’s standard deviation, which was determined by eight repeat measurements of the blank solution. It was discovered that the detection limit for FVP was 1.0 nM. A comparison of FVP detection using various sensors is presented in Table 1. According to Table 1, the prepared sensor (NiCo_2_O_4_ NPs/HMIM BF_4_/CPE) provided better performance compared to other reported sensors for FVP detection.

### 3.5. Simultaneous Detection of FVP and AC on NiCo_2_O_4_ NPs/HMIM BF_4_/CPE

**Figure 6:**
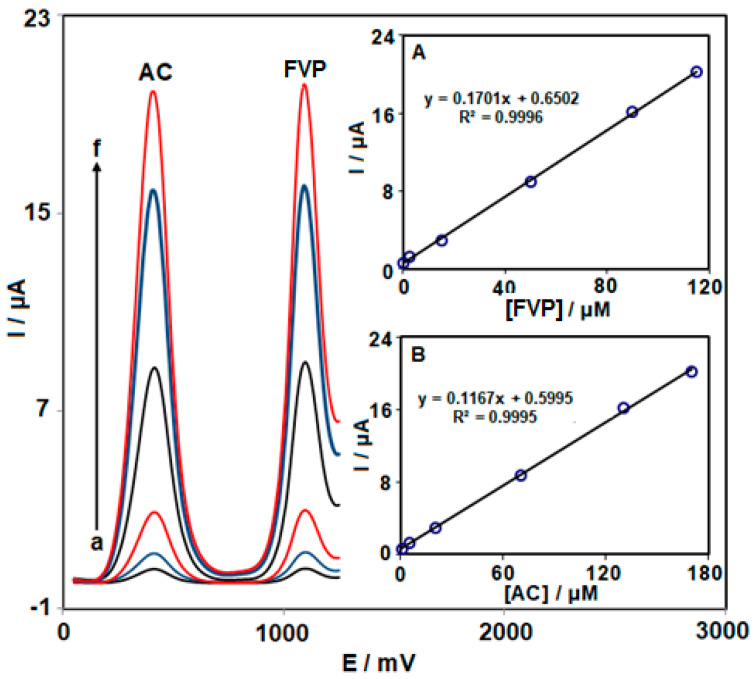
DPV response at NiCo_2_O_4_ NPs/HMIM BF_4_/CPE in 0.1 M PBS (pH = 7.0) with various concentrations of FVP (a: 0.1 µM, b: 2.5 µM, c: 15.0 µM, d: 50.0 µM, e: 90.0 µM, and f: 115.0 µM) and AC (a: 1.0 µM, b: 5.0 µM, c: 20.0 µM, d: 70.0 µM, e: 130.0 µM, and f: 170.0 µM). Insets: The corresponding plots of the anodic peak currents vs. concentrations of FVP (Inset **A**) and AC (Inset **B**).

As far as the authors’ knowledge goes, this is the first work utilizing NiCo_2_O_4_ NPs/HMIM BF_4_/CPE to detect FVP in the presence of AC. Figure 6 shows the DPVs for simultaneous FVP and AC detection using NiCo_2_O_4_ NPs/HMIM BF_4_/CPE. The oxidation of FVP and AC, respectively, was associated with the peaks at 400 and 1100 mV. By simultaneously increasing the concentrations of both analytes, the peak current intensity has been linearly increased. The matching calibration curves for FVP and AC may be seen in Figure 6 (insets A and B). The linear regression line’s slope for the calibration curve for FVP (0.1701 μAμM^−1^) has been virtually identical to that for the calibration curve without AC (0.1672 μAμM^−1^), demonstrating the viability of using NiCo_2_O_4_ NPs/HMIM BF_4_/CPE for simultaneous detection of FVP and AC concentrations.

### 3.6. Reproducibility, Repeatability and Stability of the NiCo_2_O_4_ NPs/HMIM BF_4_/CPE 

DPV was used to evaluate the reproducibility, repeatability, and stability of the NiCo_2_O_4_ NPs/HMIM BF_4_/CPE. The computed relative standard deviation (RSD) for eight measurements of 50.0 μM FVP made with a single electrode was 3.7%, showing that this sensor has acceptable repeatability.

The computed RSD for measurement of 50.0 μM FVP at five separate electrodes made in the same manner in another investigation was 2.6%, demonstrating the reasonable reproducibility of the NiCo_2_O_4_ NPs/HMIM BF_4_/CPE.

In the third investigation in this part, one modified electrode was kept for 10 days and then utilized for FVP determination to determine the stability of the NiCo_2_O_4_ NPs/HMIM BF_4_/CPE. The data show that Ip for FVP electrochemical oxidation decreased to 96.5% of its initial value after 10 days, demonstrating a high degree of stability.

### 3.7. Interference Studies

To assess the selectivity of NiCo_2_O_4_ NPs/HMIM BF_4_/CPE for FVP, an examination was carried out to determine the effect of potential interfering substances under optimized conditions. The DPV responses were recorded upon addition of interfering substances into 0.1 M PBS (pH 7.0) containing 50.0 µM FVP. DPV responses were measured by adding interfering substances to a solution of 0.1 M PBS with a pH of 7.0, containing 50.0 µM of FVP. As a general principle, the relative error in the measurement is controlled at approximately ±5% and is considered to have no interference. Also, the findings indicated that K^+^, Na^+^, NH_4_^+^, Ca^2+^, Mg^2+^, Cl^−^, Br^−^, SO_4_^2−^, NO_3_^−^, glucose, dopamine, ascorbic acid, uric acid, acetaminophen, glucose, l-cysteine and l-arginine did not interfere with the oxidation peak response of FVP.

### 3.8. Real Sample Analysis

The modified CPE was applied to pharmaceutical samples in order to evaluate the applicability of the use of a modified electrode to determine FVP and AC in real samples. The findings are provided in Table 2 using the standard addition method. Recoveries range from 96.9% to 104.8%, and relative standard deviations are all lower than or equal to 3.5%. The experimental results confirmed that the NiCo_2_O_4_ NPs/HMIM BF_4_/CPE sensor has great potential for analytical applications.

## 4. Conclusions

This study revealed the development of an electroanalytical sensor for the simultaneous detection of FVP and AC. As a result, we created an electroanalytical sensor that is amplified by NiCo_2_O_4_ NPs and HMIM BF_4_ ionic liquid. It has been discovered that the modified electrode has outstanding electrocatalytic activity for the oxidation of FVP and AC. Using the NiCo_2_O_4_ NPs/HMIM BF_4_/CPE, distinct oxidation peaks of FVP and AC have been produced, enabling the simultaneous detection of both analytes. The NiCo_2_O_4_ NPs/HMIM BF_4_/CPE is a promising candidate for FVP and AC determination in pharmaceutical samples with recovery ratios between 96.9 and 104.8% because the proposed sensor demonstrated outstanding benefits of easy preparation, strong catalytic activity, low reagent usage, and affordability. The novelty of this work is the use of NiCo_2_O_4_ NPs/HMIM BF_4_/CPE as a new sensor for the electrochemical detection of FVP in the presence of AC.

## Figures and Tables

**Table 1:** Comparison of different sensors for FVP detection.

Electrochemical Sensor	Electrochemical Technique	Linear Range	LOD	Ref.
Diamond nanoparticles/CPE	Adsorptive stripping differential pulse voltammetry	0.02–1.0 μM	4.83 nM	[84]
Platinum nanoparticles anchored on reduced graphene oxide nanocomposite/glassy carbon electrode	Square wave voltammetry	3.16–100.0 μM	2.46 μM	[85]
Au nanoparticles anchored conductive carbon black modified graphite nanopowder flakes paste electrode	Adsorptive square wave voltammetry	0.03–75 μM	7.5 nM	[86]
MnO_2_-reduced graphene oxide/screen-printed electrode	Square wave voltammetry	1.0 × 10^−8^–5.5 × 10^−5^ M	0.11 µM	[87]
Ionic liquid-carbon nanotubes/glassy carbon electrode	-	0.9–150 μM	16.0 nM	[88]
NiCo_2_O_4_ NPs/HMIM BF_4_/CPE	DPV	0.004–115.0 μM	1.0 nM	This work

**Table 2:** Estimation of FVP and AC in real samples using NiCo_2_O_4_ NPs/HMIM BF_4_/CPE (n = 5).

Sample	Spiked (μM)		Found (μM)		Recovery (%)		R.S.D. (%)	
	FVP	AC	FVP	AC	FVP	AC	FVP	AC
FVP Tablet	0	0	2.4	-	-	-	3.4	-
	2.0	5.0	4.3	5.1	97.7	102.0	2.7	2.2
	4.0	6.0	6.5	5.8	101.6	96.7	1.9	2.1
	6.0	7.0	8.8	6.8	104.8	97.1	2.3	3.5
	8.0	8.0	10.3	8.3	99.0	193.7	2.6	2.8
AC Tablet	0	0	-	3.3	-	-	-	1.7
	5.5	1.0	5.6	4.2	101.8	97.7	1.8	3.2
	6.5	2.0	6.3	5.5	96.9	103.8	3.4	2.9
	7.5	3.0	7.8	6.2	104.0	98.4	2.7	2.2
	8.5	4.0	8.4	7.4	98.8	101.4	2.4	2.6

## Data Availability

The data presented in this study are available upon request from the corresponding authors.
